# Characterization of the Insect Assemblage and Associated Floral Volatiles of Black Cherry (*Prunus serotina*)

**DOI:** 10.3390/plants10102195

**Published:** 2021-10-15

**Authors:** Craig Larcenaire, Fumin Wang, Ida Holásková, Richard Turcotte, Michael Gutensohn, Yong-Lak Park

**Affiliations:** 1 Forest Health Protection, USDA Forest Service, Morgantown, WV 26505, USA; craig.j.larcenaire@usda.gov (C.L.); richard.m.turcotte@usda.gov (R.T.); 2 Division of Plant and Soil Sciences, West Virginia University, Morgantown, WV 26506, USA; fw0003@mix.wvu.edu (F.W.); michael.gutensohn@mail.wvu.edu (M.G.); 3 Office of Statistics, West Virginia Agriculture and Forestry Experiment Station, West Virginia University, Morgantown, WV 26506, USA; ida.holaskova@mail.wvu.edu

**Keywords:** Allegheny National Forest, black cherry, Diptera, floral volatiles, Lepidoptera, pollination, *Prunus*, volatile organic compound

## Abstract

Black cherry is an ecologically important high-value wood. A decline of its regeneration has been reported in the USA, which could be associated with a lack of pollination. This study was conducted to identify insects visiting black cherry flowers, to determine whether insects captured on the flowers carry black cherry pollen and to identify the volatile organic compounds (VOCs) emitted by flowers of black cherry. A two-year insect survey was conducted before, during and after the black cherry bloom. A total of 9533 insects were captured in traps and Diptera was the most abundant (64.1%). Significantly more insects in Diptera, Lepidoptera and Thysanoptera were captured in the traps installed in the canopy than those on the ground, and *Anthalia bulbosa* (Diptera: Hybotidae) was the dominant species. Electron microscopy analyses demonstrated that insects captured in the canopy indeed carried black cherry pollen. Black cherry flowers emitted a VOC blend that is composed of 34 compounds and dominated by β-ocimene and several phenylpropanoids/benzenoids. This floral VOC profile is similar to that of other pollinator-dependent *Prunus* species. This study reports pollinator insects and associated VOCs, for the first time, that could play a significant role in the pollination and regeneration of black cherry.

## 1. Introduction

Black cherry, *Prunus serotina* (Ehrh.), is an important tree species both ecologically and economically. The growth form of the bole along with its stability and the superior working qualities of the wood make black cherry a valuable timber crop [1]. There are five subspecies and two varieties of *P. serotina* throughout North America with various morphologies [2]. The subspecies *serotina* var. *serotina* is the most common and is widely distributed throughout eastern North America [2,3]. A mature tree can grow to 20–30 m and has an average lifespan of 80–100 years [3]. Black cherry can be found growing in woodlands, thickets, roadsides and fencerows from sea level to elevations of 1500 m [1].

Ecologically, black cherry provides services to support fauna, flora and soil in the forest ecosystem. Especially in the early successional forest, black cherry provides a habitat for small mammals such as rabbits, hares, squirrels and mice [4]. The fruits of black cherry are an important source of mast for many mammals (e.g., squirrels, deer, bears and mice) and many bird species [3]. The tendency of black cherry to occupy a wide range of environments and fill disturbance gaps in the forest makes it an important stopover habitat for migrating birds [5]. In addition, black cherry is an important nectar and pollen resource for insects in forest ecosystems specifically in the early spring when other flowers are scarce [3]. Black cherry flowers are hermaphroditic (i.e., possessing both male and female reproductive organs) and self-incompatible (i.e., the inability of pollen fertilizing flowers on the same plant) [6]. The successfully cross-pollinated flowers produce a dark red to black drupe that contains a single seed.

In Europe, black cherry was introduced as an ornamental and timber species [7,8], but it has not produced the valuable timber as desired. Instead, black cherry has become an invasive species due to its ability to grow in a wide range of environments, changing the structure and function of many European forests [9]. This includes competition with native European species such as *Prunus padus*, nutrient cycling in the soil and interactions with the native insect food web [10,11]. Planting of black cherry in Europe continued throughout the 20th century for soil protection and amelioration [7] and the multiple introductions boosted the genetic diversity, which likely contributed to its adaptive success and invasive behavior [12].

Within its native range in the USA, the Allegheny Plateau in northwestern Pennsylvania is particularly well suited for the growth of high-quality black cherry [1,3,13]. However, land managers in the area have observed declining natural regeneration rates of black cherry since the end of the 20th century [14,15]. Although this decline could be caused by many factors such as stand age, deer browse, soil pathogens, ozone damage, or plant allelopathy [14,16,17,18,19,20], we have also observed a severe decline of fruit set in the area. This could be indicative of a pollination deficiency because the flowers are entomophilous and self-incompatible. The only published study involving black cherry pollinators was an observational study that was conducted by Robertson [21]. This study documented various flies, beetles and bees visiting black cherry flowers in open-grown landscape trees. In contrast, several studies suggested that honeybees and bumblebees are the primary pollinators of many other *Prunus* species in orchard settings [22]. However, there are no published data available on insects visiting black cherry flowers in forest ecosystems.

Thus, this study was conducted to characterize the insect assemblage associated with black cherry flowers and flower traits that can potentially shape the assemblage. The specific objectives of this study were: (1) to determine what insects visit the canopy and understory of black cherry stands before, during and after the flowering period; (2) to identify whether insects caught in the canopy carry black cherry pollen; and (3) to characterize black cherry flower traits such as emitted volatile organic compounds (VOCs) that potentially contribute to the attraction of insects. Specifically, we tested hypotheses in this study. First, flower-visiting insects that potentially contribute to pollination are attracted to the canopy of blooming black cherry trees and therefore are more abundant during the flowering period. Second, some of the flower-visiting insects can carry pollen on their bodies thus contributing to pollination. Third, the profile of volatiles emitted from black cherry flowers is similar to that of other pollinator-dependent *Prunus* species. Here we report for the first time on insects associated with flowering black cherry in a natural forest system and the volatile organic compounds emitted from their flowers.

## 2. Results

### 2.1. Survey and Identification of Insects Visiting Black Cherry

**Figure 1:**
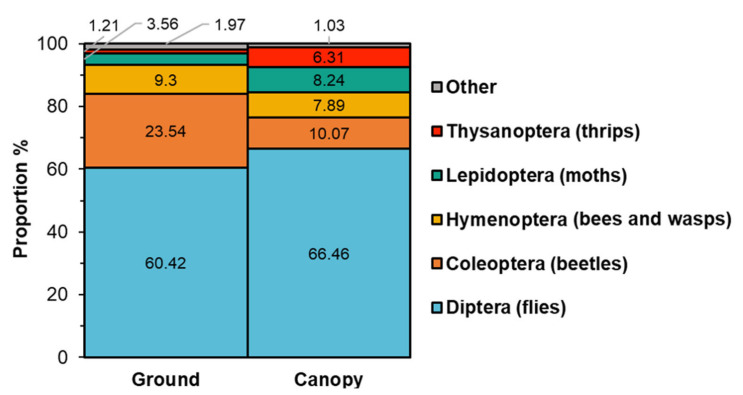
Mosaic chart showing the proportion of insect orders captured in the ground and canopy traps. The total trap captures across all trapping periods on the ground were 3878 (563 per day) and 5655 (822 per day) in the canopy.

Overall, 9533 arthropods were captured in 72 pan traps from two locations, three trees per location and two trap positions (i.e., in the canopy and on the ground) per tree, three trapping periods (i.e., before, during and after black cherry bloom) per trap over two years (2018 and 2019). Major insect orders (98% of all trap captures) were Diptera (flies), Coleoptera (beetles), Hymenoptera (bees and wasps), Lepidoptera (moths) and Thysanoptera (thrips) (Figure 1). Minor arthropods included Arachnida (spiders), Collembola (springtails), Trichoptera (caddisflies), Mecoptera (Scorpion flies), Orthoptera (grasshoppers and crickets) and Plecoptera (stoneflies). The proportions of major orders, unadjusted for trapping period and trap position, depended on the sampling year (χ^2^ = 56.4, df = 5, *p* < 0.001), with a higher proportion of Diptera (70%) in 2019 than in 2018 (56%), but a lower proportion of Lepidoptera in 2019 (4%) than in 2018 (9%). However, proportions of insect orders and sampling sites were not related (*p* > 0.05). In both trap locations across both years, Diptera consistently comprised more than 60% of the assemblage and Diptera and Coleoptera were two of the most abundant orders (Figure 1).

**Figure 2:**
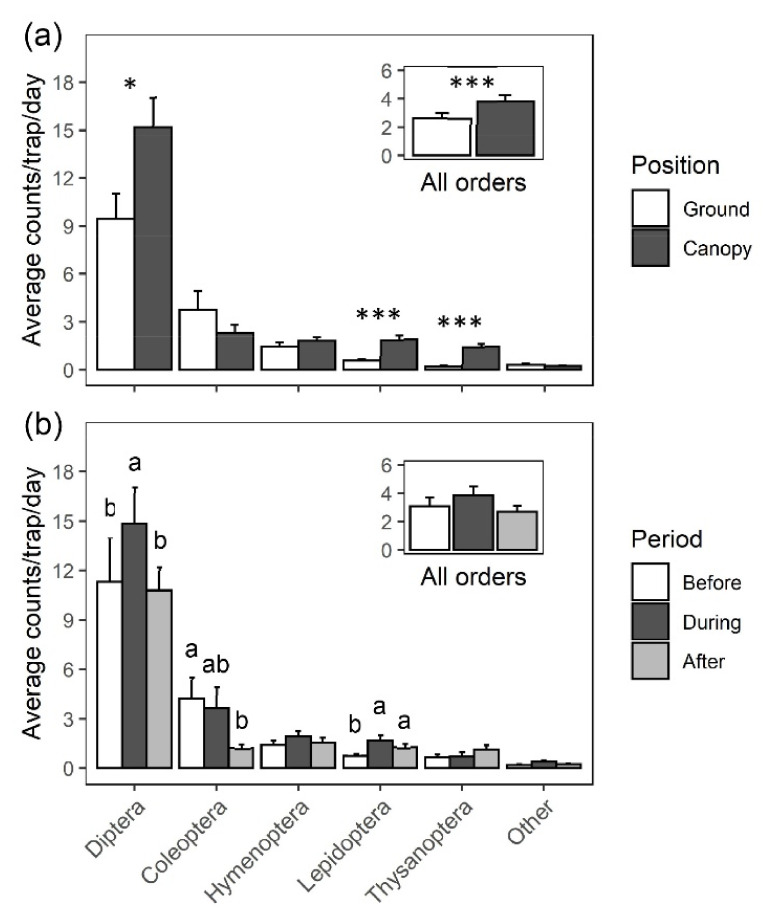
Captures of insects by orders in two trap locations (ground and canopy) and three trapping periods (before, during and after flowering): (**a**) main effect of trap position in doubly repeated measures ANOVA is indicated (α = 0.05; *, *p* < 0.05; ***, *p* < 0.001); (**b**) different letters in each insect order indicate a significant difference in trap captures among flowering periods based on Tukey–Kramer test at α = 0.05.

In the overall statistical model accounting for the sequence of flowering periods and repeated years with insect order as a random factor, we found higher captures of all arthropods in the canopy traps than on the ground (F = 11.99, *p* < 0.001) but there were no differences in trap captures among three different trapping periods (i.e., before, during and after flowering; Figure 2). Insect orders with significantly higher trap captures in the canopy traps than those in the ground traps were Diptera (F = 15.17, *p* = 0.0113), Lepidoptera (F = 32.56, *p* < 0.001) and Thysanoptera (F = 56.58, *p* < 0.001) (Figure 2). By comparing trap captures in the canopy among the three trapping periods, we found that the flowering period had a significant effect on trap captures of insects in Diptera (F = 13.92, *p* < 0.001), Coleoptera (F = 4.87, *p* = 0.02) and Lepidoptera (F = 7.1, *p* = 0.0037).

**Figure 3:**
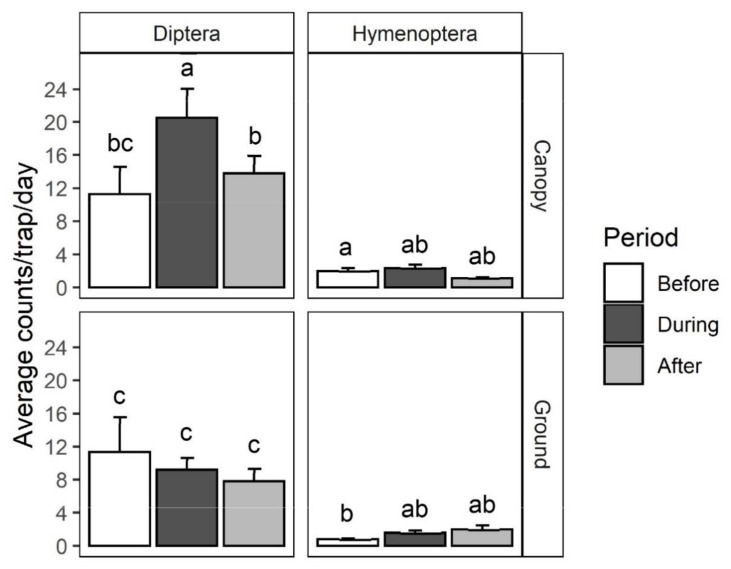
A total of 9533 insects were captured in the traps and Diptera was the most abundant (64.1%). Significantly more insects in Diptera, Lepidoptera and Thysanoptera were captured in the traps installed in the canopy than those on the ground and *Anthalia bulbosa* (Diptera: Hybotidae) was the dominant species visiting the canopy of black cherry. Different letters indicate a significant difference in trap captures at α = 0.05.

Significant interaction of trap position and flowering period was detected in Diptera (F = 5.54, *p* = 0.012) and Hymenoptera (F = 7.32, *p* = 0.004) (Figure 3). Specifically, Diptera counts in the canopy varied greatly with the flowering period; they almost doubled in number during flowering compared to before flowering (t = −5.88, *p* < 0.001), followed by a decline in numbers after flowering (t = 3.92, *p* < 0.01). However, such a pattern was not observed in the trap captures on the ground (Figure 3). During the flowering period, traps in the canopy caught significantly more dipterans than those on the ground (t = 5.13; *p* < 0.001).

Of the 5655 insects captured in the canopy, the key insect species found during the flowering period were *Anthalia bulbosa* (Diptera: Hybotidae), which comprised 11% of the total trap captures. *Frankliniella* spp. (Thysanoptera: Thripidae) were the second most abundant at 4% and *Rhamphomyia* spp., (Diptera: Empididae), *Athryglossa* spp. (Diptera: Ephydridae) and *Melanotus hyslopi* (Coleoptera: Elateridae) comprised 3%, 2% and 2% of the total trap captures, respectively (Table 1). The species with the next highest trap counts were *Eusphalerum convexum* (Coleoptera: Staphylinidae) and *Melanolophia canadaria* (Lepidoptera: Geometridae), which each comprised ~1% of the total captures. Among these major insect species, *A. bulbosa* and *Frankliniella* spp. were captured significantly more in the canopy traps than in the ground traps (Table 1).

### 2.2. Characterization of Insects Carrying Black Cherry Pollen

**Figure 4:**
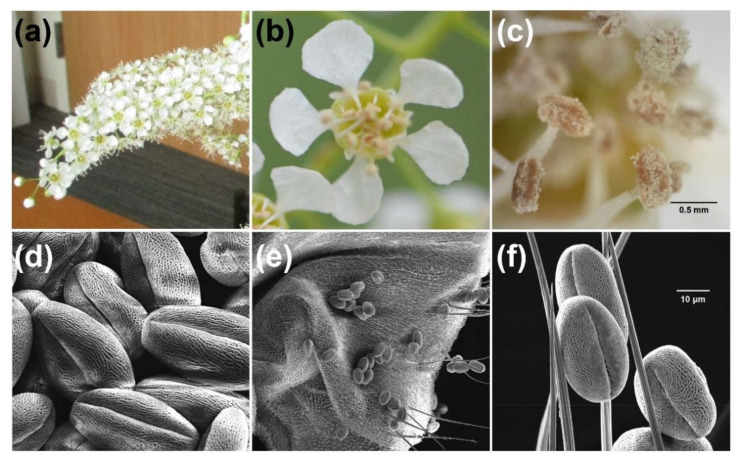
Flowers and pollen grains of black cherry: (**a**) black cherry raceme in full bloom; (**b**) a black cherry flower with 5 white petals and 14–15 stamens surrounding a single pistil; (**c**) anthers after dehiscence; (**d**) SEM image of pollen grains on anther; (**e**) SEM image of black cherry pollen grains found on the thorax of *Tipula* sp. (Diptera: Tipulidae); (**f**) black cherry pollen grains captured in insect hairs showing the unique exine sculpturing.

The pollen grains of black cherry have a distinct morphology as was revealed by scanning electron microscopy (SEM) analysis. The pollen grains have a spheroidal shape with 42.13 ± 0.58 µm (polar) by 36.83 ± 0.58 µm (equatorial) dimensions (Figure 4d). The pollen grains were found to be isopolar and tricolpate, the pollen exine was tectate and the sculpturing was striate (Figure 4d).

**Figure 5:**
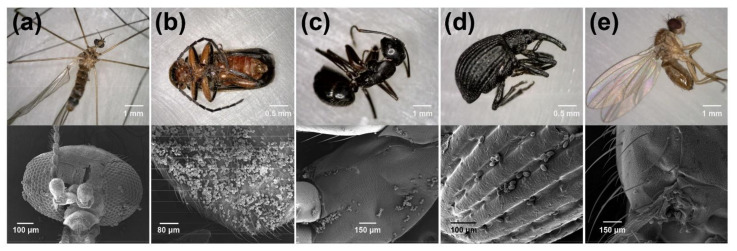
Black cherry pollen grains found on insect body: (**a**) *Antocha* sp. (Diptera: Limoniidae); (**b**) *Atalantycha bilineata* (Coleoptera: Cantharidae); (**c**) *Camponotus pennsylvanicus* (Hymenoptera: Formicidae); (**d**) *Trichopion* sp. (Coleoptera: Curculionidae); (**e**) Drosophilinae (Diptera: Drosophilidae).

Insects from three major orders (Coleoptera, Diptera and Hymenoptera) representing 12 different families were collected from black cherry flowers for SEM analysis. Each of these insects was indeed observed to be carrying pollen grains on their body (Figure 5). Electron microscopy analysis showed that the pollen on these insects (Figure 4e,f and Figure 5) matched the size, shape and exine texture of the pollen observed in the anthers of black cherry flowers (Figure 4d). A crane fly, *Antocha* sp. (Diptera: Limoniidae), was observed foraging on the flowers and was confirmed to be transporting black cherry pollen on the setae of the thorax (Figure 5a). Likewise, soldier beetles, *Atalantycha bilineata* (Coleoptera: Cantharidae), were observed foraging on the flowers and confirmed to be carrying pollen on their body (Figure 5b). All other collected insects, including the black carpenter ant, *Camponotus pennsylvanicus* (Hymenoptera: Formicidae), a weevil, *Trichopion* sp. (Coleoptera: Curculionidae) and fruit flies, *Drosophila* sp. (Diptera: Drosophilidae), were also found to be carrying black cherry pollen on their body, legs and antennae (Figure 5c–e).

### 2.3. Volatile Profile of Black Cherry Flowers

Several flower characteristics including visual traits, such as flower morphology, arrangement and pigmentation, as well as floral volatiles contribute to the attraction of pollinators. Visual traits can attract pollinators, especially when many individual flowers are arranged in larger inflorescences [23]. Individual black cherry flowers are only ~10 mm in diameter and their corolla is made up of five white petals [6] (Figure 4b). However, black cherry flowers are arranged in clusters of 30–50 individual flowers (Figure 4a) on a 10–15 cm long raceme [3]. In general, flowers emit complex and characteristic blends of volatile organic compounds (VOCs) into the surrounding atmosphere, which enables the attraction of pollinators over large distances; however, it also contributes to the defense against florivores and pathogens [24]. Our analysis of the volatile blend emitted from black cherry flowers revealed the existence of two different chemotypes among the trees in the Allegheny National Forest based on significant differences in the qualitative and quantitative composition of their floral VOC profile (Table 2, Figure S1). While 30 VOCs were emitted from flowers of both chemotypes, one and three compounds were found only in the floral volatile profile of chemotypes 1 and 2, respectively. Of the 34 floral volatile compounds observed in total, the identity of 28 could be verified by comparison with authentic standards (Figures S2–S6) and the remaining 6 compounds were tentatively identified by comparison of their mass spectra with the NIST library. The blend of volatiles emitted from black cherry flowers contained a number of monoterpenes (Table 2) with the two isomers, (*E*)- and (*Z*)-β-ocimene, together representing the most prominent of all detected volatile compounds (58.8% and 71.0% of total VOCs in chemotype 1 and 2, respectively). Other less abundant monoterpene compounds found in the floral volatile blend include α-pinene, α-myrcene, D-limonene, α-linalool, (*Z*)-linalool oxide and 3,4-dimethy, l-2,4,6-octatriene (Table 2). In contrast to the abundance and diversity of monoterpenes, only minor amounts of one sesquiterpene, (*E*,*E*)-α-farnesene, were emitted from black cherry flowers. Fatty acid derivatives are the second class of VOCs detected in the floral volatile profile of black cherry (Table 2) including the aldehydes nonanal and decanal, as well as the alkanes dodecane, tridecane, tetradecane, pentadecane, hexadecane and heptadecane. The third major group of VOCs emitted from black cherry flowers was phenylpropanoids/benzenoids (Table 2) including phenylacetaldehyde and phenylethanol, as well as benzaldehyde, methyl salicylate, methyl benzoate, ethyl benzoate and benzyl benzoate. While some of these compounds, such as benzaldehyde and phenylethanol, were produced in large quantities in flowers of chemotype 1, a different profile was observed for chemotype 2. Flowers of chemotype 2 emitted three methoxylated derivatives, *p*-anisaldehyde (4-methoxybenzaldehyde), *p*-anisyl alcohol (4-methoxybenzyl alcohol) and methyl *p*-anisate (methyl 4-methoxybenzoate), which appear to be formed at the expense of some of the other phenylpropanoids/benzenoids that were absent (phenylacetaldehyde) or formed at lower quantities (phenylethanol, benzaldehyde, methyl benzoate, benzyl benzoate). In addition to compounds of the three major VOC classes, we also found one nitrogen-containing compound, methyl nicotinate, one sulfur-containing compound, benzothiazole, and linolenic acid derived (*Z*)-jasmone in the volatile profile emitted from black cherry flowers (Table 2).

**Figure 6:**
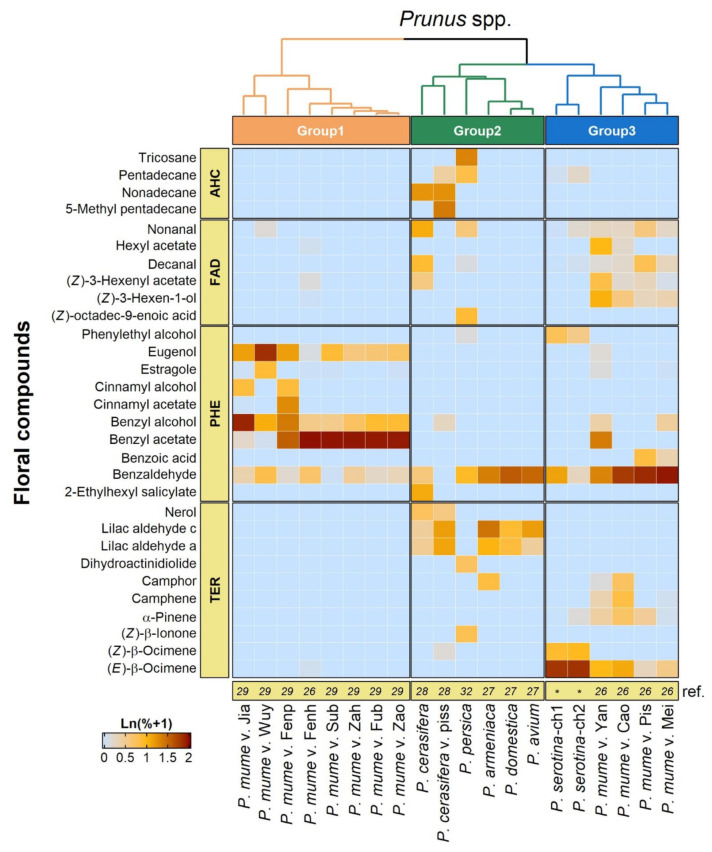
Heatmap and hierarchical clusters (groups 1–3) based on profiles of major floral volatile compounds in *Prunus serotina* and other *Prunus* species. Relative abundances (%) of volatile compounds were normalized by a shifted-log transformation, i.e., Ln (% + 1). *Prunus* species and cultivars were hierarchically clustered by Ward’s minimum variance method on Euclidean distances. Classes of floral volatile compounds: TER, terpenes and derivatives; PHE, phenylpropanoids/benzenoids; FAD, fatty acid derivatives; AHC, alkane and alkene hydrocarbons. * data reported in this study.

The *Prunus* genus contains a number of other important ornamental and fruit tree species. Since some of these *Prunus* species are highly dependent on pollinators for fruit production their floral volatile profiles have been studied previously [25,26,27,28,29,30,31,32], which allowed us to compare these with the profile observed here for black cherry (Table 2, Figure S1). Remarkably 27 of the 34 VOCs emitted from black cherry flowers were also found in the floral volatile profiles of at least one and often several other *Prunus* species (Table S1). The volatile compounds found in flowers of black cherry and other *Prunus* species belong to the three major classes terpenes, fatty acid derivatives and phenylpropanoids/benzenoids, including benzaldehyde, which were present in all studied *Prunus* species (Table S1). By hierarchical clustering of their floral volatile profiles, expressed as the relative abundance of individual VOCs, the different *Prunus* species could be assigned to three groups (Figure 6) thus further highlighting their similarity. The first group contained several cultivars of the Chinese plum (*P. mume*) and their floral volatile profiles were dominated by some phenylpropanoids/benzenoids including eugenol, benzyl alcohol and benzyl acetate, while the production of other VOCs was quite low. The second group contained various *Prunus* species, including cherry (*P. avium*), plum (*P. domestica*) and peach (*P. persica*), which are characterized by floral volatile profiles with the abundant formation of benzaldehyde and lilac aldehyde. The third group is composed of another set of *P. mume* cultivars and the two *P. serotina* chemotypes identified in this study and is characterized by (*E*)-β-ocimene and benzaldehyde as the major compounds in their floral volatile profiles.

## 3. Discussion

The Rosaceae family consists of ~100 genera and more than 3000 plant species worldwide [33]. The simple flowers in this family are considered generalists for attracting pollinators [34]. The genus *Prunus*, a member of Rosaceae [33], consists of ~200 species, many of which are economically important as orchard crops [35], including cultivated almond, peach, plum, cherry and apricot. Members of this genus typically bear five-petal flowers [36], which are self-incompatible and entomophilous. In orchards, *P. salicina* (Japanese plum) was shown to increase fruit production when managed bees were introduced to orchards [37]. Gyan and Woodell [38] analyzed pollen of *P. spinosa* (blackthorn) on *Eristalis* spp. (Diptera: Syrphidae), *Bombus* spp. (Hymenoptera: Apidae) and *Apis mellifera* (Hymenoptera: Apidae). They found that these insects transferred ample pollen to *P. spinosa*. When *Osmia cornifrons* (Hymenoptera: Megachilidae) are introduced to commercial sweet cherry (*P. avium*) orchards the trees produce larger and heavier fruit [39]. The main insect species observed pollinating peach (*P. persica*) is *A. mellifera* [22,40]. Chokecherry (*P. virginiana*) attracts bees in the genera of *Andrena* and *Bombus*, with noted observations of insects in Diptera visiting the flowers [41].

Our study conducted in the Allegheny National Forest showed a diverse assemblage of insects visiting the canopies of black cherry (Figure 1). Among this assemblage, Diptera was the most abundant group and the dominant species collected were *A. bulbosa*, *Rhamphomyia* spp., *Discocerina* spp. and *P. regina* (Table 1). We also collected native bee species that are known to be pollinators of flowers including *Auglochlora pura* (Hymenoptera: Halictidae), *Andrena carlini* (Hymenoptera: Andrenidae) and *Lasioglossum cressoni* (Hymenoptera: Halictidae). However, Hymenoptera was found in significantly lower numbers compared to Diptera in our survey. This may suggest that they do not use the flowers as often as other species, or they were not efficiently captured by the pan traps. The moth species that was most prevalent in the canopy was *Melanolophia canadaria* (Lepidoptera: Geometridae) (Table 1). This species can feed on black cherry foliage as a caterpillar and the adults have been shown to carry pollen [42]. Two of the major beetle species observed in this study, *M. hyslopi* and *E. convexum*, have not been reported as pollinators or flower visitors previously. *Anaspis rufa* (Coleoptera: Scraptiidae) is known to feed on flowers and inhabits forest ecosystems [43], which could indicate a role of this species in pollination. Although flower thrips (*Frankliniella* spp.) were the second most abundant species collected in the canopy of black cherry (Table 1), they are generally considered as florivores [44] and it remains to be shown how much they contribute to pollination. Since small insects, such as some of those observed in the black cherry canopy, are poor flyers in general and could have trouble flying long distances and in windy conditions [45,46], these insects might only cross-pollinate nearby black cherry trees.

Many angiosperms rely on insects to pollinate their flowers and thus use visual and olfactory flower cues to attract them. The flowers of *Prunus* species are similar in color and petal number, but their size and inflorescence structure are quite diverse. The flowers of black cherry are situated on racemes with 30–50 individual flowers (Figure 4) and are typically smaller (~10 mm) than those of other *Prunus* species including *P. persica* (30–40 mm), *P. spinosa* (~20 mm), *P. avium* (30–40 mm), *P. salicina* (25–50 mm) and *P. mume* (30–40 mm) [47]. In addition, these trees only grow to around 10 m in height in an open-grown orchard setting, whereas black cherry can grow to 20–30 m in natural forests. *P. virginiana* has a flower size and raceme structure comparable to black cherry; however, it represents an understory woody plant and not a canopy species. *P. padus* is similar to black cherry in flower and stem size but not raceme structure. The flowers of *P. padus* (12–15 mm each in diameter) are arranged in groups of three to seven per umbel and the bole can grow to a height of 19 m [48]. The pollinators of this species belong to six species of Diptera, two species of Hymenoptera and four species of Coleoptera [48].

While visual cues are important for the attraction of pollinators, in particular, if flowers are arranged in inflorescences that contrast against the background, floral volatiles are also considered as a crucial long-distance signal in poorly lit habitats such as forest environments [49,50]. Our analysis revealed that black cherry flowers emit a volatile blend (Table 2, Figure 1) that is primarily composed of compounds belonging to the three major classes of floral volatiles: terpenes, phenylpropanoids/benzenoids and fatty acid derivatives [24]. Based on the significant differences in the qualitative and quantitative composition of the floral volatile profiles (Table 2) we identified two black cherry chemotypes. While the floral volatile blend of chemotype 1 is more abundant in several phenylpropanoids/benzenoids including benzaldehyde, phenylacetaldehyde and phenylethanol, that of chemotype 2 is characterized by the presence of methoxylated derivatives (i.e., *p*-anisaldehyde, *p*-anisyl alcohol, methyl *p*-anisate) not found in chemotype 1. Considering the substantial genetic variation that was found within the entire eastern black cherry population in the USA [12,51,52], the identification of these two chemotypes and the potential existence of even more chemotypes are not surprising. The formation of the observed floral volatile blend composed of more than 30 VOCs (Table 2) involves multiple metabolic pathways and genes that are all potential targets for genetic variation. Similar diversity in the qualitative and quantitative composition of floral volatile profiles has recently also been observed with different cultivars of *Prunus mume* [26] (see also Figure 6) and strawberry (*Fragaria ananasa*) [32,53], another Rosaceae fruit crop.

In general, however, the majority of individual VOCs emitted from black cherry flowers (Table 2) have also been identified as floral volatiles in many other angiosperm families [54]. Remarkably, our comparison (Figure 6, Table S1) demonstrated that the floral volatile profiles of both black cherry chemotypes are very similar to that of other *Prunus* species, which are highly dependent on pollinators for fruit production. It is well known that some VOCs found in floral volatile blends contribute to the attraction of pollinators, while others are involved in the defense against florivores and pathogens [24]. However, substantial evidence has emerged from previous studies that specific VOCs, which were also found in black cherry flowers in our study, are indeed involved in the attraction of different groups of pollinators. Several of the terpenes (e.g., (*Z*)-β-ocimene, α-linalool, (*Z*)-linalool oxide, α-pinene, (*E*,*E*)-α-farnesene) and phenylpropanoids/benzenoids (e.g., phenylethanol, phenylacetaldehyde, methyl benzoate, methyl salicylate, p-anisaldehyde) emitted from black cherry flowers (Table 2) are known to be attractive to various bees (summarized in Dötterl and Vereecken [49]). Likewise, plant species that attract lepidopterans for pollination specifically release phenylpropanoids/benzenoids (e.g., phenylethanol, phenylacetaldehyde) and terpenes (e.g., linalool, linalool oxides) [55,56,57], which are also prominent in the floral volatile profile of black cherry (Table 2). Additional behavioral tests with the flower-visiting butterflies *Luehdorfia japonica* (Lepidoptera: Papilionidae) and *Pieris rapae* (Lepidoptera: Pieridae) demonstrated that a group of VOCs including phenylacetaldehyde, phenylethanol and benzaldehyde were highly attractive and elicited a respective response [30,58]. While black cherry flowers, like other *Prunus* species, clearly emit a blend of volatiles that should be attractive to Hymenoptera and Lepidoptera pollinators, surprisingly only relatively small numbers of these were observed in the canopy of black cherry trees in our survey (Figure 1). However, considering the similarly low numbers of Hymenoptera and Lepidoptera found in our ground traps (Figure 1) this appears to be due to an overall low abundance of these potential pollinators in the forest ecosystem, rather than to a lack of attraction to black cherry flowers. Although many insects in Diptera are considered as one of the most important groups of flower-visiting insects, which is in line with their high abundance in the canopy of black cherry trees observed in our surveys (Figure 1), our knowledge about their role in pollination and attraction to specific flower traits remains limited compared to the other major pollinators such as Hymenoptera and Lepidoptera. Similar to other pollinator insects, dipterans also use visual and olfactory cues to locate flowers. While some dipteran species appear to be specifically attracted to amine or sulfur-containing VOCs, many flowering plants visited by flies emit floral volatile blends that are devoid of these compounds and are rather composed of terpene, phenylpropanoid/benzenoid and fatty acid derivative volatile compounds [59]. Recent analyses [60,61,62,63] demonstrated that the antennae of flower-visiting syrphid flies are tuned to several phenylpropanoids/benzenoids (e.g., phenylacetaldehyde, phenylethanol, benzaldehyde, methyl benzoate, methyl salicylate, p-anisaldehyde) and terpenes (e.g., linalool, linalool oxides), which were all found in the floral volatile profile of black cherry (Table 2). Moreover, in field studies phenylethanol was found to be highly attractive to syrphid flies [56]. Since phenylethanol is abundant in black cherry flowers (Table 2), this suggests that this volatile compound could also contribute to the attraction of Diptera to the canopy of these trees.

In summary, this is the first report on the visitation of potential pollinators of black cherry in a natural forest ecosystem. Our data demonstrate that Diptera were the most frequently found insects in the canopy of black cherry during flowering. This suggests that these Diptera are attracted by the flower traits of black cherry, including visual traits as well as floral volatiles, and contribute to their pollination. However, due to the generalist morphology of the flowers and the similarity of the floral volatile profile to that of other *Prunus* species, it appears unlikely that a singular insect species or order, such as Diptera, is exclusively responsible for the cross-pollination of black cherry flowers. Instead, successful cross-pollination of black cherry could depend on a wide variety of opportunistic nectar and pollen feeders. The results of our insect survey need to be considered in light of the general decline in abundance and diversity of pollinating insect populations over the last decades [64,65], which might explain the underrepresentation of particular insect orders in our trap captures. The small size and weak ability to fly of the two dominant insect species observed in our surveys, *A. bulbosa* and *F. tritici*, suggests that they might not represent very efficient cross-pollinators [46]. Instead, they might primarily transport pollen within the canopy of the same tree before other pollinators could bring pollen from a different black cherry tree, which would increase instances of geitonogamy and thus prevent successful seed production.

While our study provides baseline data on the insect assemblage associated with the canopy of flowering black cherry in a natural forest ecosystem, several questions remain that require future investigations. We did not measure the correlation between seed production and the abundance/absence of specific insect species or orders. Therefore, further studies are required to verify which insects are responsible for and how much they contribute to the cross-pollination of black cherry and seed production. Colored pan traps are a widely used method to sample flower-visiting insects, but this approach is potentially biased [66,67]. These traps tend to catch honeybees, bumblebees and bees in the genus *Colletes* less frequently than expected by their perceived abundance [68]. This type of trap is also susceptible to damage caused by curious animals or certain weather conditions. Future studies with individual representative insect species performed under more controlled conditions could further verify their attraction to black cherry flowers and emitted volatiles, as well as their potential contribution to pollination. In addition, a possible decline in some pollinators (e.g., Hymenoptera and Lepidoptera) and the resulting shift in dominant insect species could explain the observed failure in fruit set and decreased natural regeneration of black cherry in recent years.

## 4. Materials and Methods

### 4.1. Survey and Identification of Insects Visiting Black Cherry

A two-year insect survey was conducted at two sites within the Allegheny National Forest in northwestern Pennsylvania, USA. The first site was located in Cherry Grove Township, Warren County (41.7238 N, −79.1242 W). The other site was ~35 km east of Cherry Grove near Bradford (41.7475 N, −78.7665 W). The stands chosen at both sites were uneven-aged mixed-species stands consisting of typical Allegheny hardwood species including hemlock (*Tsuga Canadensis*), American beech (*Fagus grandifolia*), maple (*Acer* spp.) and birch (*Betula* spp.). Each black cherry stand covered ~12 ha. Other vegetation in the stands includes raspberry (*Rubus idaeus*), blackberry (*Rubus allegheniensis*), partridgeberry (*Mitella repens*), Canada mayflower (*Maianthemum canadensis*), New York fern (*Thelypteris noveborecensis*), Trillium (*Trillium* spp.), trout lily (*Erythronium americanum*), ground pine (*Lycopodium obsurum*) and various grasses (*Poa* spp.).

To survey insect visitation to black cherry, we deployed colored pan traps from 24 May to 12 June in 2018 and 21 May to 4 June in 2019. We deployed each trap for seven days before peak flowering, during the flowering period and after peak flowering. We used pan traps for the insect survey because the canopy of black cherry formed 20–30 m above the ground and physical access to the canopy for sampling pollinators visiting flowers by hand was impossible in the dense forest setting. Three subject trees were randomly chosen in each site and two traps were deployed on each subject tree: one on the ground and one in the canopy. Each trap unit consisted of three 355 mL plastic cups (Solo, Lake Forest, IL, USA). Two of the cups were coated with fluorescent yellow and fluorescent blue paint, while the third cup was not pained, i.e., white (Figure S7a). The fluorescent-pigmented paint (Fluorescent Blue and Yellow dispersion, Guerra Paint & Pigment Corp., New York, NY, USA) was mixed with a water-based matte flexible acrylic polymer emulsion (Silica Flat, Guerra Paint & Pigment Corp., New York, NY, USA). A solution of ~25 mL unscented soap (Free and Clear Dish Soap, Seventh Generation, Burlington, VT, USA) per 3.8 L of water was used to fill the cups [69]. These three colors and the trap design have been shown to attract different orders of insects [70,71]. The canopy traps, consisting of the three cups placed on a platform made from plastic pail lids, were hung in the crown of the selected black cherry trees using a slingshot (Big Shot Slingshot, Sherrill Tree, Greensboro, NC, USA) and paracord rope (Figure S7b,c). For comparison with the trap captures in the canopy, the ground traps were positioned directly below the canopy traps and placed on 30 cm wooden stakes. The insects caught in the traps were strained from the soap solution using a fine mesh paint strainer and stored in sample bags with 70% ethanol. All insect samples were sorted and identified to family and morphospecies using a stereomicroscope (Olympus SZ71, Olympus Inc., Tokyo, Japan) equipped with a digital camera (Olympus DP21, Cell Sens Dimension, Olympus Inc., Tokyo, Japan). Major insect species found in the samples were further identified to species with the help of insect taxonomists: Robert Acciavatti (Coleoptera), Andrea Kautz (Diptera), Sam Droege (Hymenoptera) and Gwan-Seok Lee (Thysanoptera).

For data analysis, trap captures from the three colored cups were combined and treated as a single trap unit. Trap counts were normalized by dividing the total number of captures by the number of days of trap deployment. The lack of normal distribution of residuals on normalized averaged trap counts was compensated by taking the square root of the normalized averaged counts (i.e., $\sqrt{x+0.0001}$). Three separate statistical analyses were conducted to determine the effects of trap location and flowering period on trap capture. First, the proportions of trap captures among insect orders were analyzed by Cochran-Mantel-Haenszel (CMH) frequency analysis that tested whether or not insect orders were related to the position of the trap (i.e., ground and canopy), flowering periods (i.e., before, during and after flowering), years (i.e., 2018 and 2019) and sampling sites (i.e., Bradford and Cherry Grove sites). Second, effects of trap positions, flowering periods and sampling years and their interactions on trap captures were analyzed across all insect orders using doubly repeated measures ANOVA [72]. Repeated factors were the year and flowering period, by using unstructured and compound symmetry covariance structure, respectively. The insect order was used as a random effect and least-square means were compared using Tukey–Kramer adjustment. Individual analyses for specific insect orders were also conducted using doubly repeated measures ANOVA as described above. Lastly, the effect of the trap position on counts of major insect species during the flowering period was examined using the Wilcoxon (rank sums) test followed by Chi-square approximation. All the data analyses were conducted with SAS 9.4. and JMP Pro 14.0 [73] and significance criterion α for all tests was 0.05.

### 4.2. Characterization of Insects Carrying Black Cherry Pollen

To determine whether insects visiting black cherry carried its pollen, we collected additional insect samples and black cherry flowers from a site in Morgantown, WV, USA (39.6465 N, −79.8794 W). For pollen sampling, a black cherry tree with a widespread canopy easily assessable from the ground was selected. Five branches with flower buds were cut from the tree before the onset of anthesis and immediately placed in a bucket with water. To capture flower visitors, a 50 mL centrifuge tube was carefully placed over insects visiting the flowers. Both insect and flower samples were transported to the laboratory for further observation and analysis.

To characterize black cherry pollen morphology, sampled flowers were observed until anthers opened to release pollen. The newly opened anthers were removed and coated with gold (200–400 Å in thickness) using a Denton Desk V sputter coater (Dentonvacuum LLC) [74]. The morphology of black cherry pollen and its exine structure were examined using SEM (S-4700, Hitachi, Tokyo, Japan) at the Shared Research Facilities of West Virginia University and photographed with the SEM beam condition set at 5.0 kV and 10 µA. The SEM images were used to determine the shape, size and exine structure of the pollen grains. The insects collected from black cherry flowers were prepared and analyzed by SEM using the protocol described above. The morphological characteristics and exine structure of pollen grains found on these insects were then compared to those of pollen grains collected from the anthers of black cherry flowers.

### 4.3. Collection and Analysis of Floral Volatiles

Branches from black cherry trees located in the Allegheny National Forest were sampled during full anthesis. Cut branches were placed into a water-filled container and kept at a stable temperature for transport. Volatiles emitted from black cherry flowers were collected using a closed-loop stripping method as described previously [75,76]. Five racemes or sections of racemes with open flowers were cut from freshly harvested branches for each volatile collection. Headspace collections from detached racemes supplemented with 20% (*w/v*) sucrose solution were performed for 24 h using Porapak-Q traps (Volatile Collection Trap LLC, Gainesville, FL, USA). Subsequently the Porapak-Q traps were eluted with dichloromethane and 3.33 µg of naphthalene was added as internal standard.

Samples from headspace collections were analyzed by combined gas chromatography/mass spectrometry (GC/MS) using a TRACE 1310 gas chromatograph system linked to a TSQ 8000 Triple Quadrupole mass spectrometer (Thermo Fisher Scientific, Waltham, MA, USA) as described previously [75,76]. Individual compounds were identified using the Xcalibur 2.2 SP1.48 software (Thermo Fisher Scientific) by comparing their mass spectra with those deposited in the NIST/EPA/NIH Mass Spectral Library (NIST11) (National Institute of Standards and Technology NIST, Scientific Instrument Services, Inc., Ringoes, NJ, USA; https://chemdata.nist.gov/mass-spc/ms-search/; accessed on 24 March 2021). The identity of compounds was confirmed by the comparison of retention times and mass spectra with authentic standards (Table S2). These standards also allowed the determination of response factors, which were used in combination with the internal standard for the quantification of analyzed compounds.

We also investigated how the profile of volatiles emitted from black cherry flowers differs from respective profiles described previously for closely related *Prunus* species [25,26,27,28,29,30,31,32,35]. The quantities of the floral volatile compounds in each *Prunus* species were converted to percentages and their major volatile compounds emitted (>4%) were assembled in a database. Subsequently, the profiles were all normalized by “shifted log” transformation, compared by a hierarchical clustering analysis (Ward’s minimum variance method) and visualized by a “Heatmap” function in “ComplexHeatmap” package [77] in R 3.6.3. In addition, the quantities of each volatile compound found in the two chemotypes were compared by using *t*-tests with α = 0.05.

## Figures and Tables

**Table 1:** Major insect species captured per day (± SE) in the canopy and ground traps during the flowering period (α = 0.05; *, *p* < 0.05; **, *p* < 0.01).

Order	Family	Species	Canopy	Ground	χ^2^	*p* Value
Diptera	Hybotidae	*Anthalia bulbosa*	109.7 ± 1.9	31.5 ± 1.6	7.2445	0.0071 **
Thysanoptera	Thripidae	*Frankliniella* spp.	40.2 ± 0.6	6.2 ± 0.4	4.083	0.0433 *
Diptera	Empididae	*Rhamphomyia* spp.	30.4 ± 1.1	11.3 ± 0.7	1.899	0.1682
Diptera	Ephydridae	*Discocerina* spp.	20.8 ± 1.0	1.7 ± 0.3	1.547	0.2136
Coleoptera	Elateridae	*Melanotus hyslopi*	16.5 ± 0.4	1.4 ± 0.1	0.7259	0.3942
Coleoptera	Staphylinidae	*Eusphalerum convexum*	14.5 ± 1.3	0.9 ± 0.1	1.0891	0.2967
Lepidoptera	Geometridae	*Melanolophia canadaria*	9.14 ± 0.9	0.7 ± 0	3.3251	0.0682
Coleoptera	Scraptiidae	*Anaspis rufa*	8.4 ± 0.3	1 ± 0.1	1.1136	0.2913
Hymenoptera	Halictidae	*Auglochlora* spp.	5.7 ± 0.1	3.5 ± 0.2	1.7526	0.1855
Coleoptera	Chrysomelidae	*Crepidodera violacea*	4.3 ± 0.2	0.6 ± 0	1.3125	0.2519
Diptera	Calliphoridae	*Phormia regina*	3.4 ± 0.1	0.1 ± 0	0.3333	0.5637

**Table 2:** Volatile organic compounds identified in the headspace of *Prunus serotina* flowers.

	Compound ^1^	CAS ^2^	NIST RI ^3^	Exp RI ^4^	Chemotype 1	Chemotype 2	*p* Value ^5^
					Pmol/Flower/Hr (Mean ± SE, *n* = 5)		
1	α-Pinene	80-56-8	937	935	0.24 ± 0.1	1.3 ± 0.69	0.1683
2	Benzaldehyde	100-52-7	962	962	161.21 ± 37.4	4.27 ± 0.62	0.0030 **
3	α-Myrcene	123-35-3	991	993	3.16 ± 0.38	2.23 ± 0.34	0.1011
4	D-Limonene	138-86-3	1030	1031	10.75 ± 2.41	3.19 ± 0.6	0.0159 *
5	*(Z*)-β-Ocimene	3338-55-4	1038	1041	56.88 ± 8.41	31.23 ± 7.14	0.0486 *
6	Phenylacetaldehyde	122-78-1	1045	1047	15.49 ± 3.19	-	0.0012 **
7	(*E*)-β-Ocimene	3779-61-1	1049	1054	415.91 ± 67.99	230.1 ± 43.54	0.0503
8	(*Z*)-Linalool oxide	5989-33-3	1074	1090	0.7 ± 0.16	1.54 ± 0.97	0.4139
9	Methyl benzoate	93-58-3	1094	1098	13.73 ± 2.25	3.5 ± 1.86	0.0080 **
10	α-Linalool	78-70-6	1099	1104	4.59 ± 0.52	3.36 ± 1.08	0.3327
11	Nonanal	124-19-6	1104	1107	1.46 ± 0.35	2.65 ± 1.26	0.3857
12	Phenylethanol	60-12-8	1116	1119	71.51 ± 10.02	15.66 ± 3.47	0.0007 ***
13	*3,4-Dimethyl-2,4,6-octatriene*	57396-75-5	1121	1132	0.98 ± 0.23	0.11 ± 0.11	0.0092 **
14	Methyl nicotinate	93-60-7	1139	1142	3.33 ± 0.78	13.31 ± 2.69	0.0073 **
15	Ethyl benzoate	93-89-0	1171	1175	2.91 ± 0.41	1.18 ± 0.33	0.0104 *
16	Methyl salicylate	119-36-8	1192	1199	2.75 ± 0.2	0.12 ± 0.12	<0.001 ***
17	Dodecane	112-40-3	1200	1200	0.02 ± 0.02	2.07 ± 1.05	0.0853
18	*Decanal*	112-31-2	1206	1209	0.71 ± 0.17	0.86 ± 0.3	0.6788
19	*N-Phenylformamide*	103-70-8	1221	1225	6.1 ± 2.13	6.56 ± 1.36	0.8585
20	Benzothiazole	95-16-9	1229	1232	4.79 ± 1.53	6.55 ± 1.47	0.4303
21	*p*-Anisaldehyde	123-11-5	1250	1264	-	14.73 ± 3.23	0.0019 **
22	*p*-Anisyl alcohol	105-13-5	1290	1293	-	6.89 ± 1.92	0.0070 **
23	Tridecane	629-50-5	1300	1300	0.39 ± 0.09	1.66 ± 0.86	0.1839
24	*N,N-Dibutylformamide*	761-65-9	1310	1308	0.54 ± 0.08	0.56 ± 0.13	0.8589
25	*Texanol*	77-68-9	1380	1381	0.76 ± 0.15	1.82 ± 0.97	0.3075
26	Methyl *p*-anisate	121-98-2	1373	1383	-	3.36 ± 0.64	0.0007 ***
27	Tetradecane	629-59-4	1400	1400	0.52 ± 0.12	0.78 ± 0.3	0.4336
28	(*Z*)-Jasmone	488-10-8	1394	1406	7.93 ± 1.42	3.18 ± 0.82	0.0199 *
29	Pentadecane	629-62-9	1500	1500	0.7 ± 0.22	0.35 ± 0.05	0.1648
30	(*E*,*E*)-α-Farnesene	502-61-4	1508	1514	4.97 ± 0.53	0.95 ± 0.22	<0.001 ***
31	Hexadecane	544-76-3	1600	1600	1.21 ± 0.37	1.94 ± 0.62	0.3388
32	*4-sec-Butyl-2,6-di-tert-butylphenol*	17540-75-9	1640	1650	6.91 ± 3.06	1.32 ± 0.9	0.1175
33	Heptadecane	629-78-7	1700	1700	0.98 ± 0.24	0.62 ± 0.12	0.2151
34	Benzyl benzoate	120-51-4	1762	1789	2.24 ± 0.45	0.16 ± 0.13	0.0022 **

^1^ Compounds highlighted in italic are only identified by comparison of mass spectra with the NIST library. ^2^ Chemical Abstract Service (CAS) registry numbers listed as numerical identifiers of chemical compounds. ^3^ Median values of retention indices for semi-standard non-polar columns (obtained from NIST/EPA/NIH MS library version 2.2). ^4^ Experimental retention indices relative to C8-C24 n-alkane standards on TraceGOLD TG-5MS GC column according to the Van den Dool–Kratz equation. ^5^ Compounds were compared between the two chemotypes by unpaired *t*-tests (α = 0.05; *, *p* < 0.05; **, *p* < 0.01; ***, *p* < 0.001).

## Data Availability

The data that support the findings of this study will be available upon request.

## Supplementary Materials

The following are available online at https://www.mdpi.com/article/10.3390/plants10102195/s1. Figure S1: Characterization of the profile of volatile organic compounds emitted from black cherry flowers. Volatiles were analyzed by GC/MS and total ion chromatograms are shown for both chemotypes. Compounds were identified based on their mass spectra and retention time: 1–34, see Table S2 for compound identity; IS, internal standard (naphthalene); Figure S2: Confirmation of VOC identity by comparison of volatiles emitted from black cherry flowers with authentic terpene standards. Volatiles and standards were analyzed by GC/MS and total ion chromatograms are shown for: floral volatiles (A,H), α-pinene (B), α-myrcene (C), D-limonene (D), ocimene isomers (E), linalool oxide isomers (F), α-linalool (G), farnesene isomers (I); Figure S3: Confirmation of VOC identity by comparison of volatiles emitted from black cherry flowers with authentic phenylpropanoid/benzenoid standards. Volatiles and standards were analyzed by GC/MS and total ion chromatograms are shown for: floral volatiles (A,G), benzaldehyde (B), phenylacetaldehyde (C), methyl benzoate (D), phenylethanol (E), ethyl benzoate (F), benzyl benzoate (H); Figure S4: Confirmation of VOC identity by comparison of volatiles emitted from black cherry flowers with authentic standards of methoxylated aromatic compounds. Volatiles and standards were analyzed by GC/MS and total ion chromatograms are shown for: floral volatiles (A), *p*-anisaldehyde (B), *p*-anisyl alcohol (C), methyl *p*-anisate (D); Figure S5: Confirmation of VOC identity by comparison of volatiles emitted from black cherry flowers with authentic standards of fatty acid derivative compounds. Volatiles and standards were analyzed by GC/MS and total ion chromatograms are shown for: floral volatiles (A), nonanal (B), hexadecane (C), alkane standard C8–C20 (D); Figure S6: Confirmation of VOC identity by comparison of volatiles emitted from black cherry flowers with authentic standards of other volatile compounds. Volatiles and standards were analyzed by GC/MS and total ion chromatograms are shown for: floral volatiles (A), methyl nicotinate (B), methyl salicylate (C), benzothiazole (D), (*Z*)-jasmone (E); Figure S7: Ground (a) and aerial (b,c) pan traps with three different colors: white, blue and yellow; Table S1: Floral volatiles identified in *Prunus serotina* and other *Prunus* species; Table S2: Volatile organic compounds used as authentic standards for the verification and quantification of compounds observed in black cherry flowers.
