# Supplementary material for: Characterization of the Insect Assemblage and Associated Floral Volatiles of Black Cherry (Prunus serotina)
Source: Plants (Basel). 2021 Oct 15;10(10):2195. doi: 10.3390/plants10102195 (PMC8538322; doi:10.3390/plants10102195)
Supplement: Supplementary file 1 [file plants-10-02195-s001.zip › plants-1402933-supplementary.pdf]

Table S1 Floral volatiles identified in *Prunus serotina* and other *Prunus* species

Compound	CAS	*P. serotina*		*P. mume*[2]	*P. mume*[5]	*P. avium*[3]	*P. persica*[8]	*P. domestica*[1]	*P. cerasifera*[4]	*P. padus*[1]	*P. yedoensis*[6]	*P. mahaleb*[7]
		Chemotype 1	Chemotype 2	Chinese plum	Chinese plum	cherry	peach	plum	cherry plum	bird cherry	Yoshino cherry	Mahaleb cherry
		relative amount %										
α-Pinene	80-56-8	0.03	0.35	0 - 4.30								
Benzaldehyde	100-52-7	20.04	1.16	4.10 - 84.90	0.14 - 5.83	30.80	7.95	4.50	0 - 3.2	19.80	32.67	0.20
α-Myrcene	123-35-3	0.39	0.61	0 - 0.80								
D-Limonene	138-86-3	1.34	0.87	0 - 0.60	0 - 0.11				tr			0.30
(*Z*)-β-Ocimene	3338-55-4	7.07	8.48						0 - 0.6			
Phenylacetaldehyde	122-78-1	1.93					0.40	0.10		0.30	0.13	
(*E*)-β-Ocimene	3779-61-1	51.71	62.51	0.20 - 11.50								0.50
(*Z*)-Linalool oxide	5989-33-3	0.09	0.42					tr	tr - 1.3	0.70		0.30
Methyl benzoate	93-58-3	1.71	0.95	0 - 0.40	tr - 1.51	1.50		tr		tr		
α-Linalool	78-70-6	0.57	0.91	0 - tr		11.40		tr	1.2		0.02	4.10
Nonanal	124-19-6	0.18	0.72	0 - 3.10	tr - 0.58		3.20	0.10	0 -10.8			1.9
Phenylethanol	60-12-8	8.89	4.25				0.51	tr		15.10	1.10	0.5
Ethyl benzoate	93-89-0	0.36	0.32					0.10				
Methyl salicylate	119-36-8	0.34	0.03	0.20 - 1.40	tr - 0.12	1.50			0 - tr			
Dodecane	112-40-3	0.00	0.56					tr				
Decanal	112-31-2	0.09	0.23	0 - 5.40			0.41		0 - 7.4			
p-Anisaldehyde	123-11-5		4.00					0.20			0.18	0.7
p-Anisyl alcohol	105-13-5		1.87					0.30			0.04	
Tridecane	629-50-5	0.05	0.45					tr				0.2
Methyl *p*-anisate	121-98-2		0.91			2.80					0.02	
Tetradecane	629-59-4	0.06	0.21				1.67					
(*Z*)-Jasmone	488-10-8	0.99	0.86									0.3
Pentadecane	629-62-9	0.09	0.10				5.93		0 - 1.8			0.2
(*E*,*E*)-α-Farnesene	502-61-4	0.62	0.26			2.80			tr - 1.8	0.10		
Hexadecane	544-76-3	0.15	0.53				3.34		0 - 0.9			0.5
Heptadecane	629-78-7	0.12	0.17				1.63		1.1 - 1.6			0.8
Benzyl benzoate	120-51-4	0.28	0.04		tr - 1.33					tr		

Floral volatile data for different Prunus species originated from the following publications: [1] Radulovic et al. (2009); [2] Hao et al. (2014); [3] El-Sayed et al. (2018); [4] Reidel et al. (2017); [5] Zhang et al. (2020); [6] Omura et al. (1999); [7] Mastelic et al. (2006); [8] Kang & Xu (2008).

Table S2 Volatile organic compounds used as authentic standards for the verification and quantification of compounds observed in black cherry flowers

Compound	CAS	Provider	Order Number
Terpenes			
α-Pinene	80-56-8	Sigma-Aldrich	147524
α-Myrcene	123-35-3	Sigma-Aldrich	64643
D-Limonene	138-86-3	Acros Organics	179395000
Ocimene (mix of isomers)	3338-55-4	Sigma-Aldrich	W353901
Linalool oxide (mix of isomers)	5989-33-3	Sigma-Aldrich	62141
Linalool	78-70-6	Acros Organics	125151000
Farnesene (mix of isomers)	502-61-4	Sigma-Aldrich	W383902
Phenylpropanoids			
Benzaldehyde	100-52-7	Sigma-Aldrich	GSK5067
Phenylacetaldehyde	122-78-1	Sigma-Aldrich	W287407
Methyl benzoate	93-58-3	Sigma-Aldrich	18344
Phenylethanol	60-12-8	Sigma-Aldrich	77861
Ethyl benzoate	93-89-0	Sigma-Aldrich	E12907
Benzyl benzoate	120-51-4	Sigma-Aldrich	68183
p-Anisaldehyde	123-11-5	Sigma-Aldrich	97063
p-Anisyl alcohol	105-13-5	Sigma-Aldrich	136905
Methyl *p*-anisate	121-98-2	Sigma-Aldrich	253146
FADs			
Nonanal	124-19-6	Sigma-Aldrich	442719
Hexadecane	544-76-3	Tokyo Chemical Industry	H0066
Alkane Standard (C_8-C_{20})		Sigma-Aldrich	04070
Other VOCs			
Methyl nicotinate	93-60-7	Sigma-Aldrich	M59203
Methyl salicylate	119-36-8	Sigma-Aldrich	M6752
Benzothiazole	95-16-9	Sigma-Aldrich	101338
cis-Jasmone	488-10-8	Sigma-Aldrich	W319600

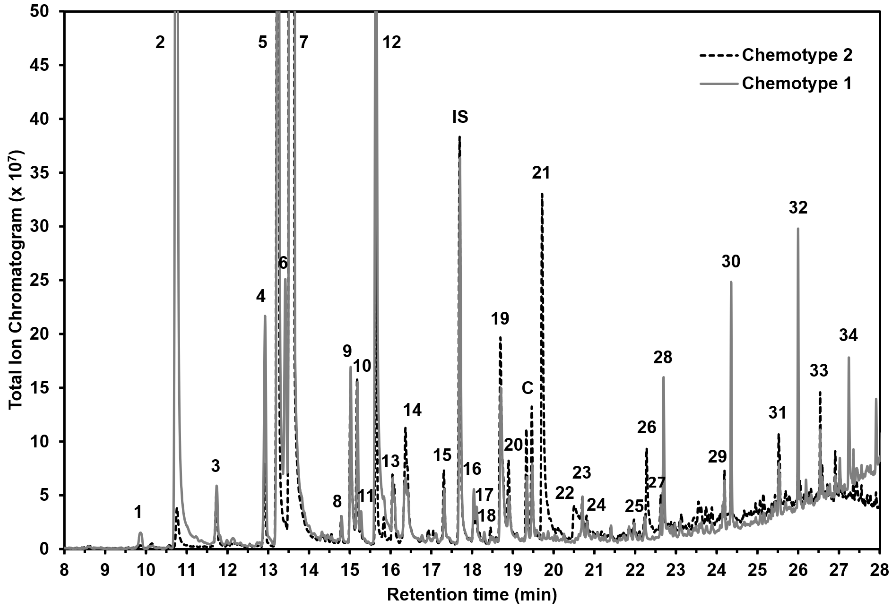

Figure S1 Characterization of the profile of volatile organic compounds emitted from black cherry flowers. Volatiles were analyzed by GC/MS, and total ion chromatograms are shown for both chemotypes. Compounds were identified based on their mass spectra and retention time: 1 - 34, see Table 2 for compound identity; IS, internal standard (naphthalene).

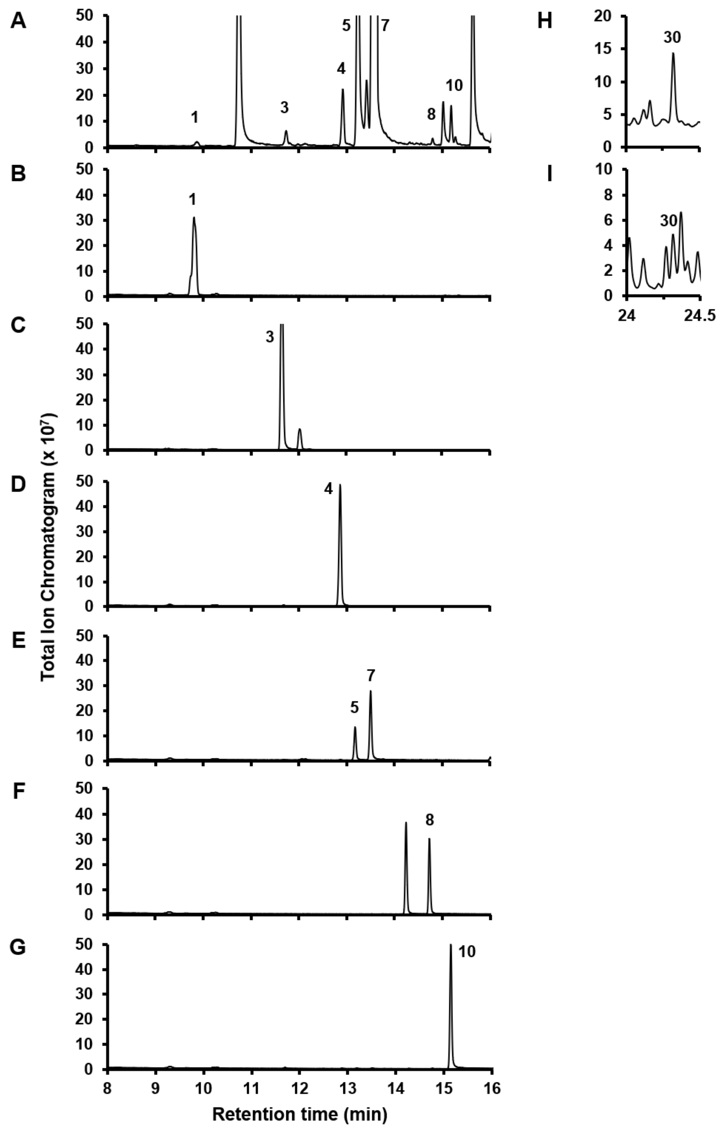

Figure S2 Confirmation of VOC identity by comparison of volatiles emitted from black cherry flowers with authentic terpene standards. Volatiles and standards were analyzed by GC/MS, and total ion chromatograms are shown for: floral volatiles (A & H), α-pinene (B), α-myrcene (C), D-limonene (D), ocimene isomers (E), linalool oxide isomers (F), α-linalool (G), farnesene isomers (I).

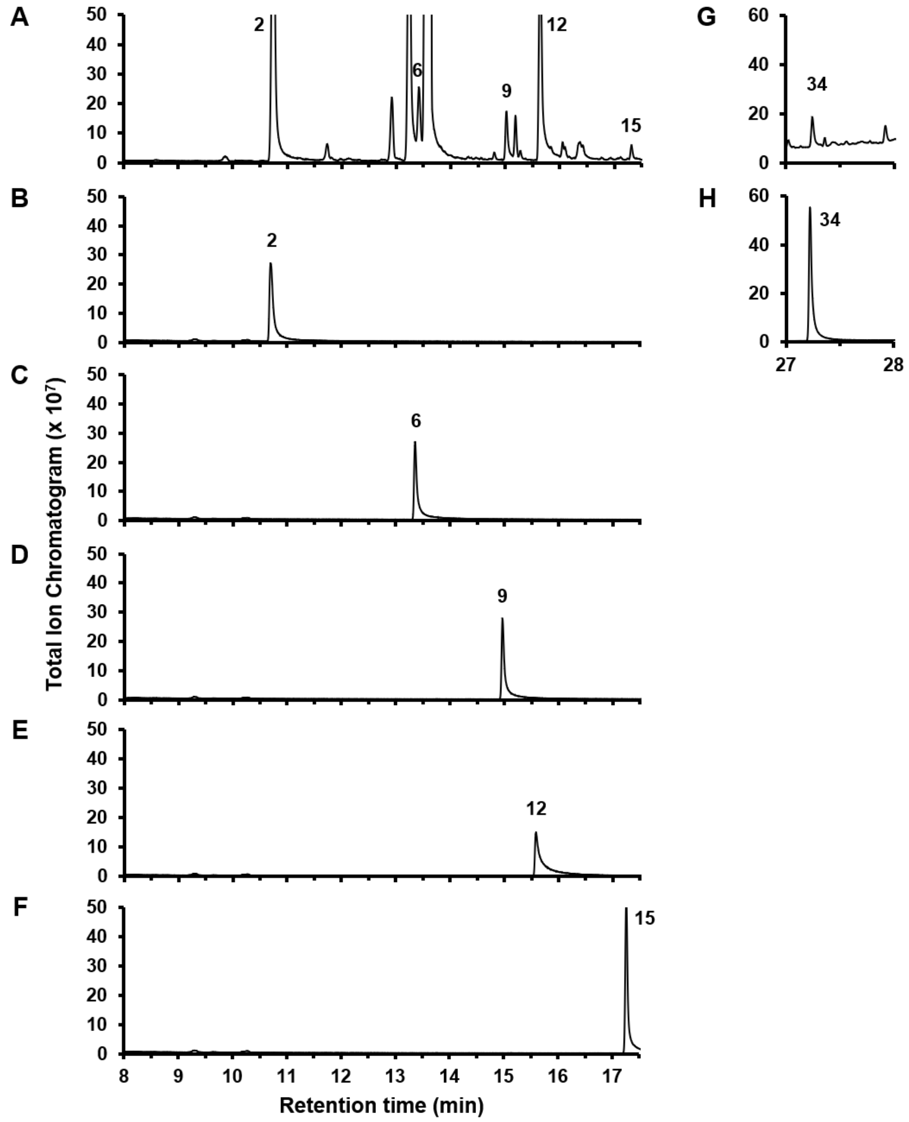

Figure S3 Confirmation of VOC identity by comparison of volatiles emitted from black cherry flowers with authentic phenylpropanoid/benzenoid standards. Volatiles and standards were analyzed by GC/MS, and total ion chromatograms are shown for: floral volatiles (A & G), benzaldehyde (B), phenylacetaldehyde (C), methyl benzoate (D), phenylethanol (E), ethyl benzoate (F), benzyl benzoate (H).

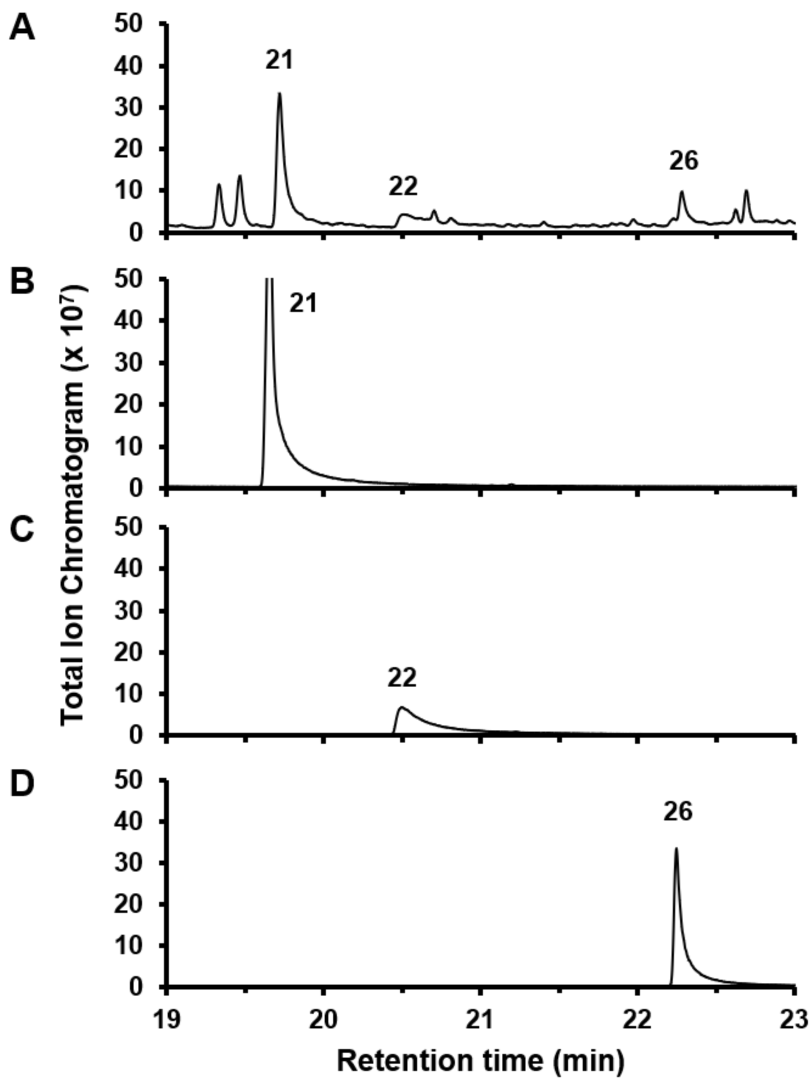

Figure S4 Confirmation of VOC identity by comparison of volatiles emitted from black cherry flowers with authentic standards of methoxylated aromatic compounds. Volatiles and standards were analyzed by GC/MS, and total ion chromatograms are shown for: floral volatiles (A), *p*-anisaldehyde (B), *p*-anisyl alcohol (C), methyl *p*-anisate (D).

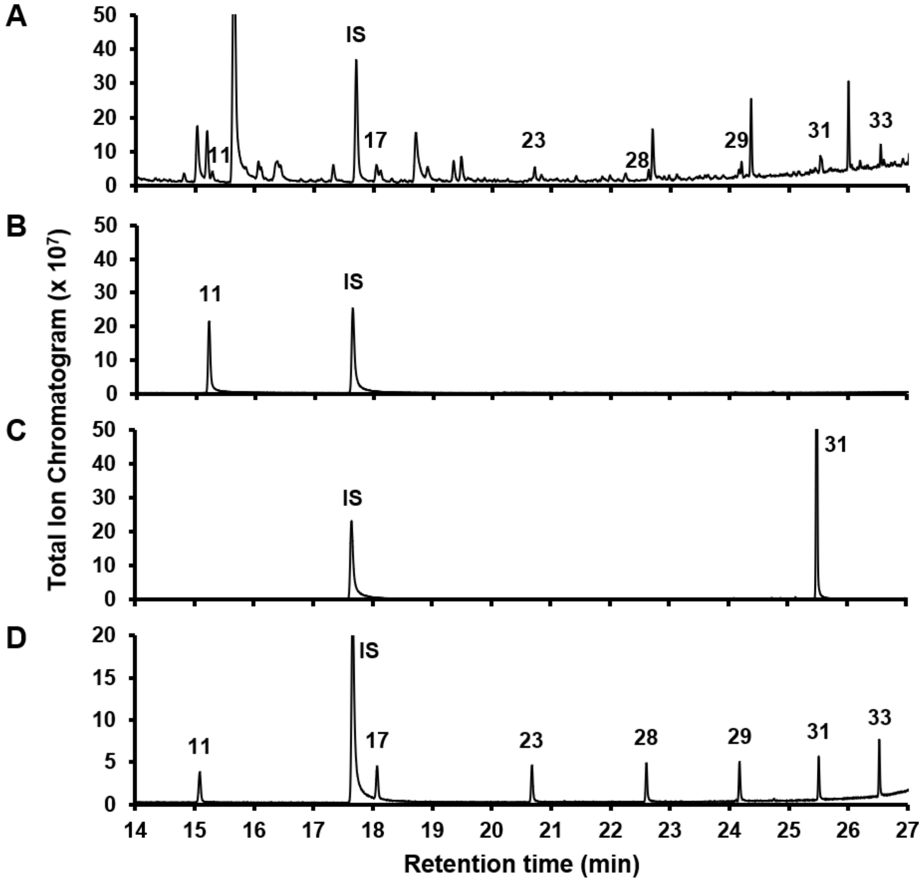

Figure S5 Confirmation of VOC identity by comparison of volatiles emitted from black cherry flowers with authentic standards of fatty acid derivative compounds. Volatiles and standards were analyzed by GC/MS, and total ion chromatograms are shown for: floral volatiles (A), nonanal (B), hexadecane (C), alkane standard C8 - C20 (D).

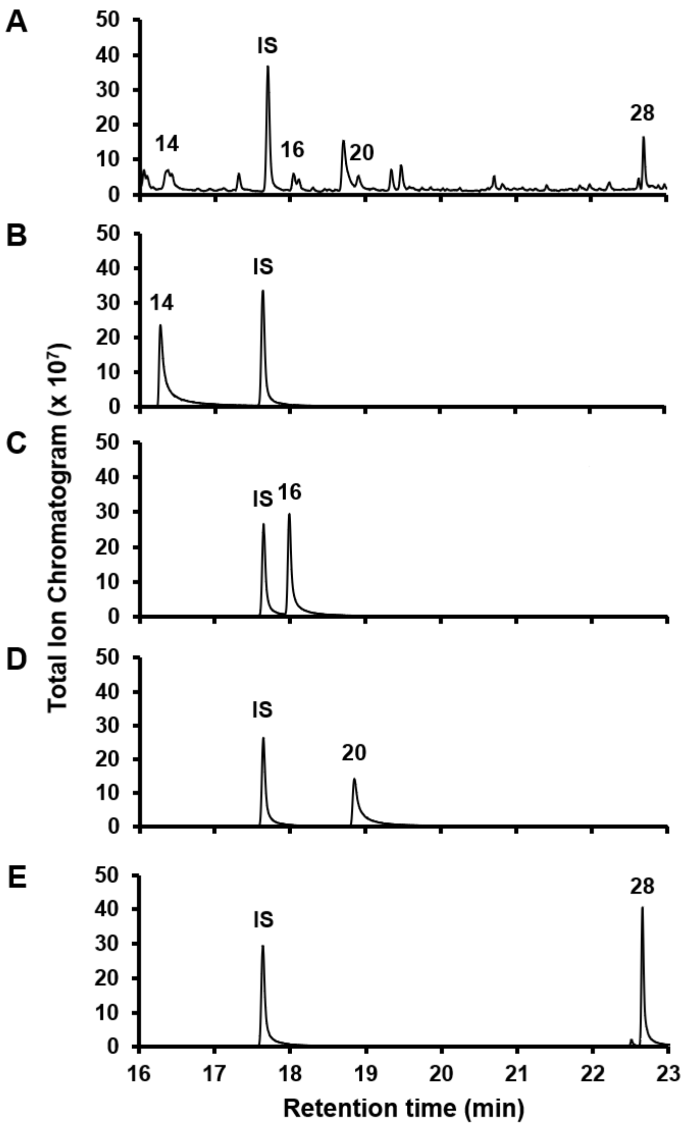

Figure S6 Confirmation of VOC identity by comparison of volatiles emitted from black cherry flowers with authentic standards of other volatile compounds. Volatiles and standards were analyzed by GC/MS, and total ion chromatograms are shown for: floral volatiles (A), methyl nicotinate (B), methyl salicylate (C), benzothiazole (D), *cis*-jasmone (E).

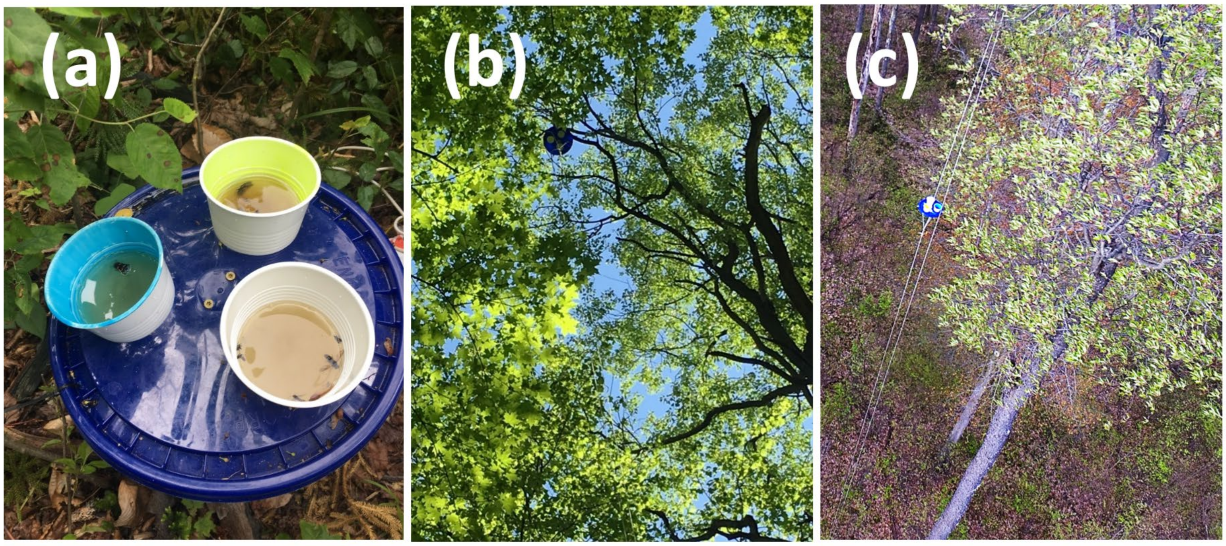

Figure S7 Ground (a) and aerial (b and c) pan traps with three different colors: white, blue, and yellow.
